# A Software Tool Aimed at Automating the Generation, Distribution, and Assessment of Social Media Messages for Health Promotion and Education Research

**DOI:** 10.2196/11263

**Published:** 2019-05-07

**Authors:** Katja Reuter, Alicia MacLennan, NamQuyen Le, Jennifer B Unger, Elsi M Kaiser, Praveen Angyan

**Affiliations:** 1 Department of Preventive Medicine, Keck School of Medicine of University of Southern California Institute for Health Promotion & Disease Prevention Research University of Southern California Los Angeles, CA United States; 2 Southern California Clinical and Translational Science Institute Keck School of Medicine University of Southern California Los Angeles, CA United States; 3 Linguistics Department Psycholinguistics Lab University of Southern California Los Angeles, CA United States

**Keywords:** algorithm, automation, digital, Facebook, health communication, health promotion, Instagram, internet, online, smoking, social network, social media, tobacco, Twitter

## Abstract

**Background:**

Social media offers promise for communicating the risks and health effects of harmful products and behaviors to larger and hard-to-reach segments of the population. Nearly 70% of US adults use some social media. However, rigorous research across different social media is vital to establish successful evidence-based health communication strategies that meet the requirements of the evolving digital landscape and the needs of diverse populations.

**Objective:**

The aim of this study was to expand and test a software tool (Trial Promoter) to support health promotion and education research by automating aspects of the generation, distribution, and assessment of large numbers of social media health messages and user comments.

**Methods:**

The tool supports 6 functions (1) data import, (2) message generation deploying randomization techniques, (3) message distribution, (4) import and analysis of message comments, (5) collection and display of message performance data, and (6) reporting based on a predetermined data dictionary. The tool was built using 3 open-source software products: PostgreSQL, Ruby on Rails, and Semantic UI. To test the tool’s utility and reliability, we developed parameterized message templates (N=102) based upon 2 government-sponsored health education campaigns, extracted images from these campaigns and a free stock photo platform (N=315), and topic-related hashtags (N=4) from Twitter. We conducted a functional correctness analysis of the generated social media messages to assess the algorithm’s ability to produce the expected output for each input. We defined 100% correctness as use of the message template text and substitution of 3 message parameters (ie, image, hashtag, and destination URL) without any error. The percent correct was calculated to determine the probability with which the tool generates accurate messages.

**Results:**

The tool generated, distributed, and assessed 1275 social media health messages over 85 days (April 19 to July 12, 2017). It correctly used the message template text and substituted the message parameters 100% (1275/1275) of the time as verified by human reviewers and a custom algorithm using text search and attribute-matching techniques.

**Conclusions:**

A software tool can effectively support the generation, distribution, and assessment of hundreds of health promotion messages and user comments across different social media with the highest degree of functional correctness and minimal human interaction. The tool has the potential to support social media–enabled health promotion research and practice: first, by enabling the assessment of large numbers of messages to develop evidence-based health communication, and second, by providing public health organizations with a tool to increase their output of health education messages and manage user comments. We call on readers to use and develop the tool and to contribute to evidence-based communication methods in the digital age.

## Introduction

The use of social media (ie, social networks or social networking sites) as a health promotion and intervention tool provides new opportunities and challenges for both investigators and practitioners [1-6]. Social media includes widely accessible Web-based and mobile technologies that allow users to view, create, and share information online and to participate in social networking [7]. On the basis of previous research, these tools offer promise for communicating with larger and hard-to-reach segments of the population and for purposes as diverse as the provision of health information, delivery of behavior change interventions, disease monitoring and self-management, awareness raising, and advocacy [8-11]. Research in other fields showed that social media messaging could have a significant impact on user attitudes and behavior. In 2017, we saw experimental evidence that these tools can be deployed as engines for social manipulation and to influence voting in elections [8,9]. However, there is limited evidence as to whether social media can support the delivery of targeted and personalized behavior change interventions to improve health [10], partly owing to the challenges of implementing large-scale social media communication experiments.

Today, nearly 70% of US adults use some social media [11]. Among the most popular platforms are Facebook, YouTube, Pinterest, Instagram, Twitter, LinkedIn, and Snapchat [12]. Their user base varies by demographic characteristics such as age, gender, and race and ethnicity [11,13]. The success of digital health communication efforts might, therefore, not only depend on the type of content (eg, text, image, audio, and video) but also on variables such as the type of social media platform, organic messages versus paid (advertisements), the message date and time, and users’ social networks. However, researchers and public health agencies typically lack the resources and expertise to disseminate and test the effectiveness of larger numbers of health messages on social media, and the majority of current interventions are neither evidence-based nor widely adopted [14]. Rigorous research across different social media types will be required to establish successful evidence-based health communication strategies that meet the requirements of the evolving digital landscape and the needs of diverse and vulnerable populations.

The objective of this study was to expand and test a software tool (Trial Promoter) to support health promotion and intervention research by automating aspects of the generation, distribution, and assessment of large numbers of health messages and user comments across different social media. In this study, we have described the tool including the features that support rigorous scientific study design such as randomization and the use of a data dictionary. The tool builds on our previous study where we demonstrated that a software tool has the ability to support increased output of research information on Twitter while reducing the burden of developing and distributing hundreds of individual messages [15] and that such an automated approach provides a cost-effective solution to distribute clinical trial information more efficiently [16].

## Methods

### Overview Description of the Technical Framework and Dataflow

**Figure 1:**
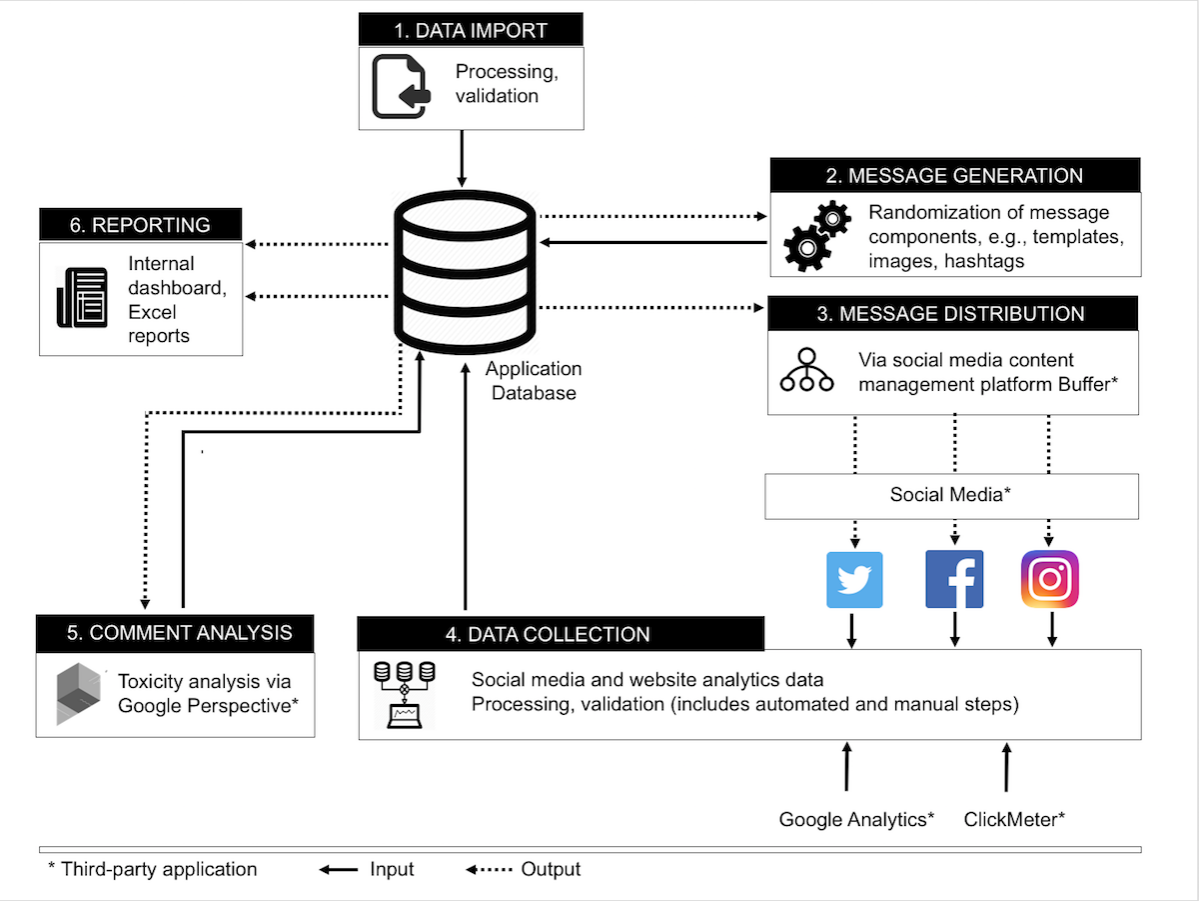
Application setup and data flow diagram.

The software tool, Trial Promoter, supports 6 functions (Figure 1): (1) data import (eg, parameterized message templates and images), (2) message generation deploying randomization techniques to reduce selection bias for message templates and characteristics (eg, images and hashtags), (3) message distribution across social media (the current version supports Twitter, Facebook, and Instagram), (4) collection of message and website performance data, (5) import of message comments and their toxicity score (ie, probability between 0 to 1, with higher values indicating a greater likelihood of offensive, disrespectful language), and (6) display of message performance data in the internal dashboard and output-reporting based on the predetermined data dictionary.

The tool is built using 3 open-source software products: the PostgreSQL object-relational database (version 9.3) [17], the *Ruby on Rails* Web framework (version 4.2.6) [18], and the Semantic UI frontend framework (version 2.2.11) [19]. Semantic UI supports the creation of dashboards and front-end interfaces. We further used the following infrastructure: the cloud application platform, *Heroku*, to deploy the tool quickly without the need to set up servers or install software [20] and *Amazon Web Services* to store image assets [21].

### Supported Data Sources, Formats, and Types

The tool is capable of importing information from different types of data sources, that is, Representational State Transfer (REST) Application Programming Interface (API), and data files. Data formats include JavaScript Object Notation (JSON), Comma Separated Values (CSV), Excel, Portable Network Graphics, and Joint Photographic Experts Group image formats. Social media messages consist of different elements such as message text, URL, hashtags, images, and videos. The current version of the tool can import all of these data types except video files.

### Data Import, Processing, and Validation

The tool offers a standard template for importing data such as message templates, social media platforms to be used, hashtags, destination URLs, and experiment-specific variables such as disease terms or linguistic factors. During import, the data included in the import file are processed in 3 ways: (1) to associate imported images with message templates for randomization, (2) to associate experiment variables (eg, disease terms and linguistic factors) with a message template, and (3) to add message template parameters for the destination URL. The tool performs data validation after the import is complete. The study team can view any validation errors detected by the tool and fix message templates or images as needed. At present, the tool carries out 2 main validation steps during the setup of the experiment: (1) it verifies that the length of the message templates for the Twitter platform fits within platform limits (ie, 280 character limit) while taking into account the length of destination URLs, image URLs, and inclusion of hashtags [22] and (2) it checks the resolution of images to meet the requirements on the social media platform, Instagram (Multimedia Appendix 1) [23]. For example, if the length of a message template does not allow for the inclusion of all hashtags from a preexisting set, the tool flags the message template as not suitable so that the study team can make the necessary adjustments.

### Setting Up Experiment Parameters

The tool supports scientific study design methods by providing options for experiment customization (Multimedia Appendix 2). An experiment is defined as a set of parameters that are used to characterize a specific health communications study, for example, to examine and compare the influence of different linguistics methods used in the messages (eg, perspective taking, information packaging, and numeracy). The present version of the tool provides the following parameters for customizing an experiment, that is, name of experiment, start date, social media platforms to be used, medium (advertised messages/advertisements vs organic, nonpaid messages), image inclusion, message repetition (ie, the number of times a message should be sent), the number of messages per day, social media accounts to be used, time schedule for message distribution, and tracking when a user clicks on a message link.

### Randomization

To reduce bias in the distribution of message characteristics, the tool randomizes elements such as message templates, images, and hashtags. The message templates were shuffled into a random sequence using a Fisher-Yates shuffle [24], and the selection of hashtags and the images were randomly sampled [25].

### Message Generation

**Figure 2:**
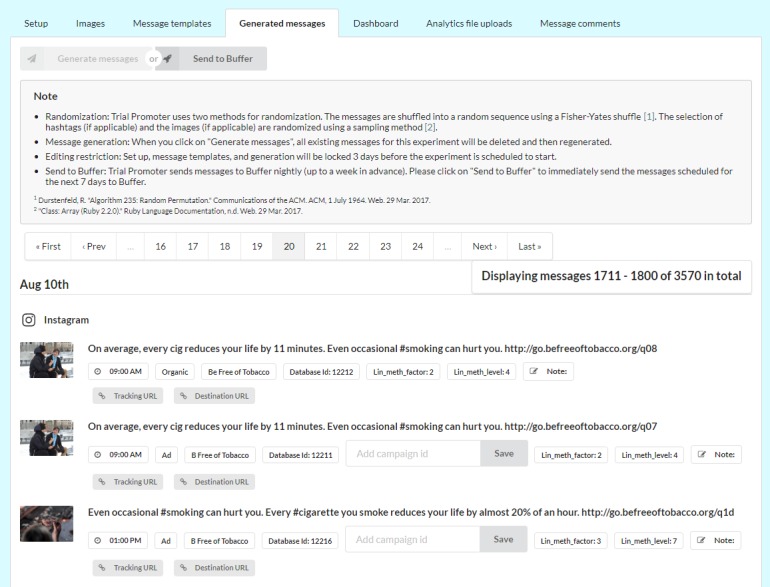
Screenshot of interface with the final messages the application generated for the correctness analysis described in this paper. Images shown here are samples similar to the original copyright protected campaign images and courtesy of Pixabay.com.

Through automatic substitution of 3 message parameters (ie, images, hashtags, and destination URLs) in the message templates, the tool generates the final messages for each social media platform (ie, Twitter, Facebook, and Instagram; Figure 2). Filled-in parameters include the destination URL to the respective Web page (ie, landing page) and a randomly chosen hashtag from a preexisting set for those messages that do not already include a hashtag (eg, #tobacco and #smoking). The message URL is tagged with Urchin Traffic Monitor parameters to track the engagement with the message on social media, that is, to track clicks on the URL that takes users to the landing page. The tool uses the REST API provided by the third-party service, Clickmeter, to generate the shortened URL. The generation of the final messages is locked once the distribution of the messages begins to prevent inadvertent changes to the messages or their deletion.

### Message Distribution

The tool schedules and distributes the messages through the project-related social media accounts using the third-party application, *Buffer*, a social media content management Web application [26]. Each social media account that is set up in *Buffer* has a unique profile identifier assigned to it. The tool allows each experiment to specify which social media accounts are to be used. *Buffer* provides a REST API call that allows for queuing of messages directly in *Buffer*. *Buffer* then sends the messages to multiple social media platforms at the scheduled times that were entered during the experiment setup.

### Data Collection and Processing

Analytics for each message to determine the engagement among social media users with the message and on the referred to Web page were collected using a number of applications that are summarized in Multimedia Appendix 3. Message comments need to be collected manually by logging into the respective social media account.

### Reporting

The application provides 2 methods of reporting: (1) project-internal dashboards accessible via login and (2) reports for statistical analysis. For each experiment, the app supports the creation of a data dictionary, which centralizes the information about the data to be collected using experiment-specific data definitions (eg, value names, meaning, origin, and format) to generate customized and comprehensive reports. See Multimedia Appendix 4 for an example of a data dictionary.

**Figure 3:**
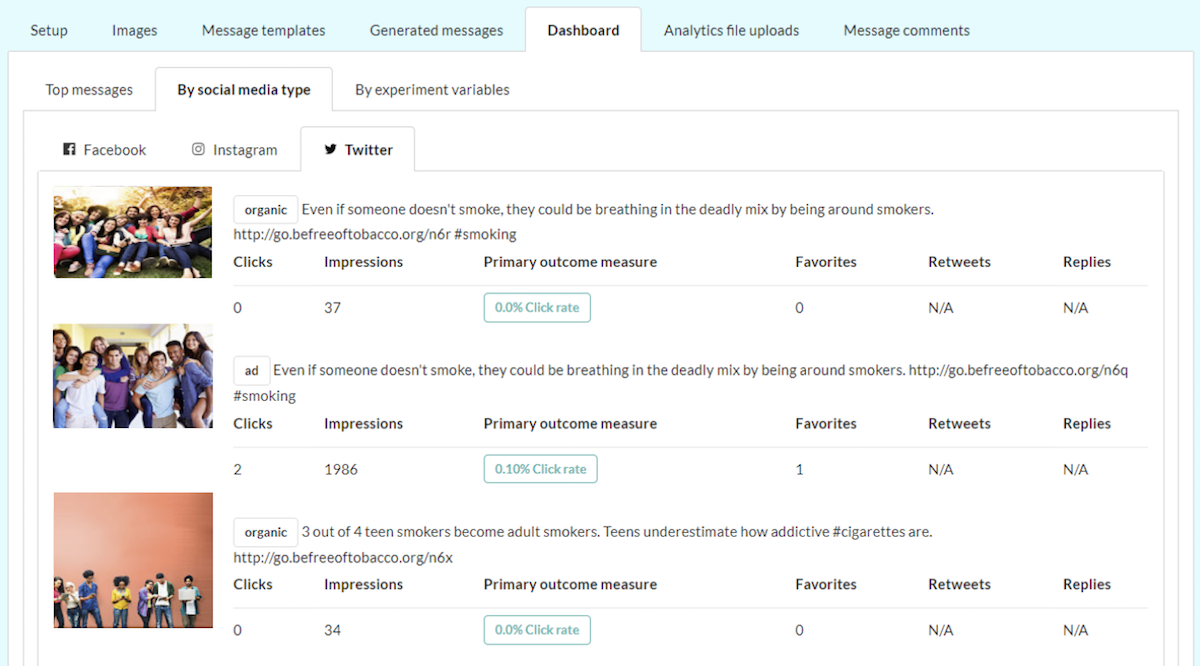
Screenshot shows dashboard interface where the application displays key performance data for each individual message by social media type. Images shown here are samples similar to the original copyright protected campaign images. Images are courtesy of Apomares (top), Monkey Business Images (middle), Rawpixel at FreeDigitalPhotos.net (bottom).

**Figure 4:**
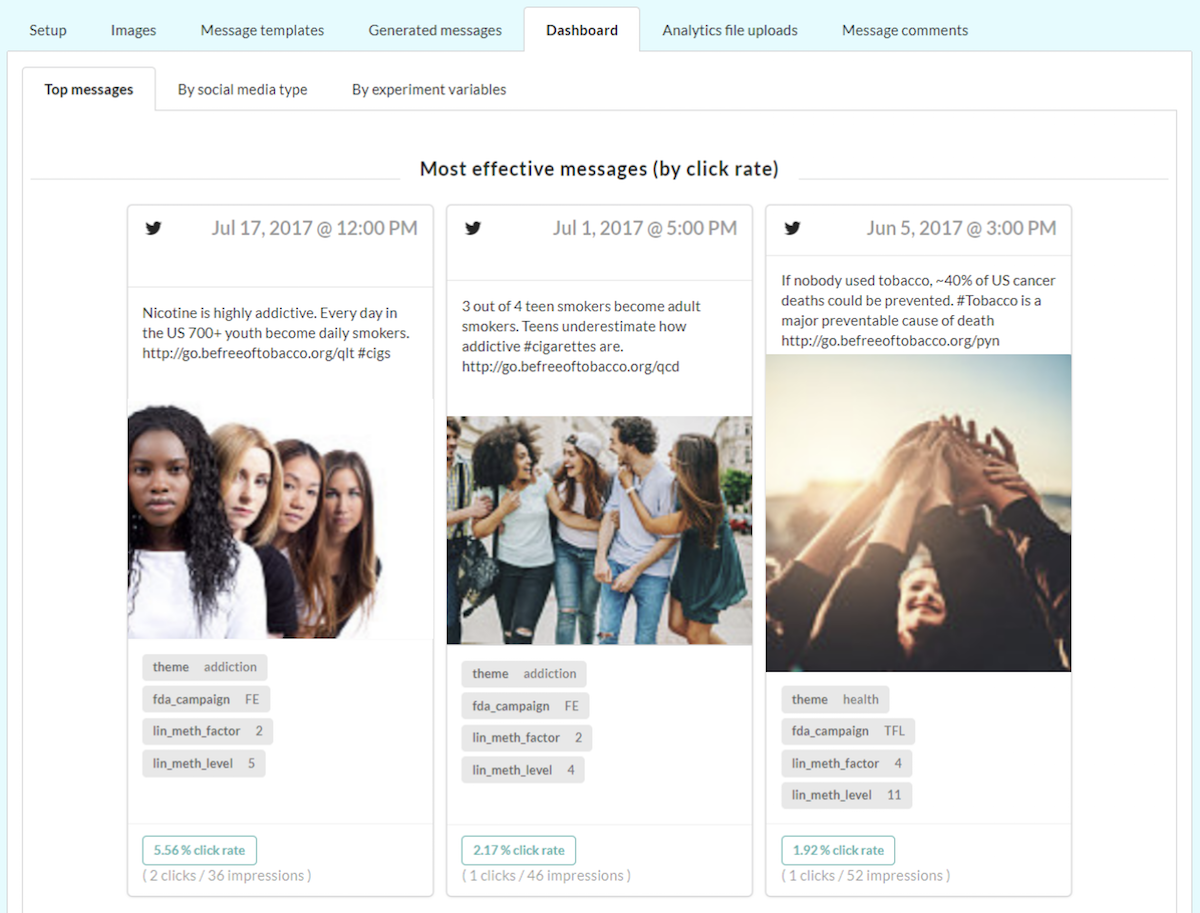
Screenshot shows dashboard interface where the application displays messages with the highest click rate. Images shown here are samples similar to the original copyright protected campaign images. Images are courtesy of Absolut Images (left), Pixelfit (middle), Aleksandar Georgiev at FreeDigitalPhotos.net (bottom).

**Figure 5:**
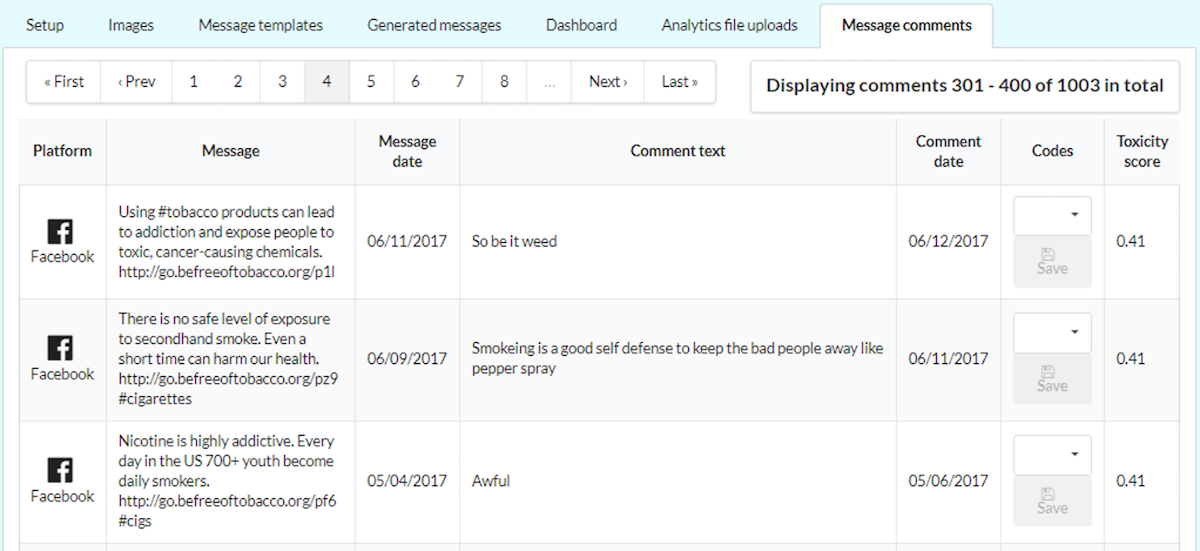
Screenshot shows dashboard interface where the application displays the comments received in response to the messages on Facebook and their toxicity score. The messages with the highest toxicity scores are listed first.

In the internal dashboard, the application provides 3 types of data visualization: (1) key performance data (eg, clicks, impressions, and click rate) for each individual message by social media type (Figure 3), (2) messages with the highest click rate (number of clicks divided by number of impressions; Figure 4), and (3) comments received in response to the messages and their toxicity score (Figure 5).

### Comment Analysis

The tool has the capability to import data (ie, comments) into the machine learning tool, *Perspective*, developed by *Jigsaw* under the umbrella of *Google* ’s parent company, *Alphabet* [27,28]. *Perspective* then calculates the toxicity score for each imported comment and determines the probability of a comment being labeled by human moderators as *toxic*. Higher values of a toxicity score between 0 to 1 indicate a greater likelihood of offensive, disrespectful language that could negatively impact an online conversation. The machine learning model used by *Perspective* is based on crowdsourced annotations of randomly sampled comments from the body of 63 million comments from the English Wikipedia [29]. Human annotators were given a scale for how likely an online participant would leave a conversation owing to the perceived abuse (very toxic, toxic, neither, healthy contribution, and very healthy contribution) [30]. The toxicity scores generated by *Perspective* are then returned in the JSON format and parsed, and the toxicity score is stored for each comment within the application.

Finally, the application provides reports, for example, for statistical analysis, in CSV format. The data reports are customizable and include the data specified for a particular experiment (ie, no filtering is applied to the data). The reports are tailored to reflect the data dictionary that was specified for the experiment and can be generated on the back-end of the application using a Rails console, an application that allows programmers to interact with the system from a command line interface, directly issuing commands that are interpreted and executed by the system.

### Correctness Analysis

To test the tool’s utility and reliability, we developed parameterized message templates (N=102) based upon 2 government-sponsored online tobacco education campaigns and extracted images from these campaigns and the free stock photo platform, Stocksnap (N=315), and extracted topic-related hashtags (N=4) from Twitter. The daily message volume per social media platform was 6 on Facebook (advertisements and organic), 6 on Twitter (advertisements and organic), and 3 on Instagram (advertisements only, owing to the fact that Instagram does not support referral URLs in organic, nonpaid messages). Advertisements and organic messages were sent to separate accounts during this experiment. The daily message volume can be customized manually during the experiment setup. It is not limited but it is recommended to stay within the social media platform–specific limit to avoid flagging or shutdown of an account, for example, the limit on Twitter is 1000 direct messages per day and 2400 tweets per day [31]. We further recommend taking into account market research data that suggest the optimal number of posts per day, for example, on Twitter, it is 3 [32,33]. During this experiment, the tool sent 3 messages per social media account at different times. The length of the pilot project (85 days) was determined by the available budget for social media advertisements. On the basis of market research showing that messages sent at these times receive the most user engagement [32-34], the tool sent messages on Facebook at 9 am, 1 pm, and 3 pm PST; on Twitter at noon, 3 pm, and 5 pm PST; and on Instagram at 8 am, 9 am, and 5 pm PST.

We conducted a functional correctness [35] analysis of the automatically generated social media messages that were distributed across the 3 social media, Twitter, Facebook, and Instagram.

We assessed the algorithm’s ability to produce the expected output for each input and defined 100% correctness as the correct use of the message template text and correct substitution of 3 message parameters (ie, image, hashtag, and destination URL). For example, an error constitutes a missing image, a missing or misspelled and therefore nonfunctional URL, or a missing or misspelled hashtag. The percent that was correct was calculated to determine the probability with which the tool generates accurate messages.

## Results

### Evaluation

**Figure 6:**
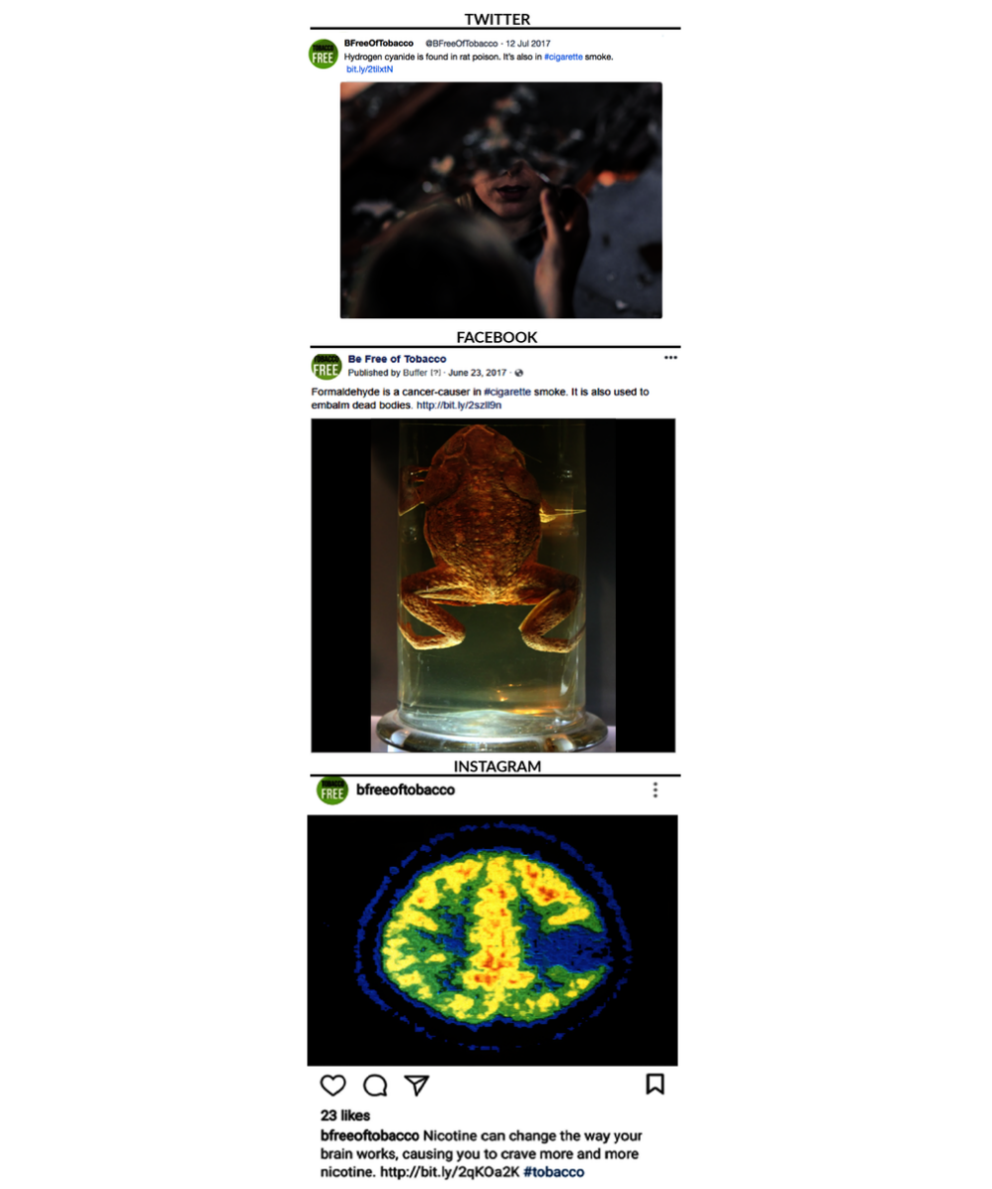
Examples of automatically generated messages that were distributed by the application across Twitter, Facebook, and Instagram. Images shown here are samples similar to the original copyright protected campaign images. Images are courtesy of Pixabay.com (top), Anagoria at Wikimedia.org (middle), Mary Bates at Wikimedia.org(bottom).

During the 85-day experiment between April 19 and July 12, 2017, the tool successfully generated and distributed a total of 1275 messages (Twitter: N=510; Facebook: N=510; and Instagram: N=255). Figure 6 shows examples of automatically generated and distributed messages that were part of the correctness analysis described here. The software code of the application is accessible under the MIT license on GitHub [36]. The detailed analysis of the messages and influence of several variables on user attention and engagement will be discussed in a forthcoming paper.

### Correctness Analysis

The correctness with which the application generated the social media messages during the experiment was evaluated using 3 factors for each individual message: (1) the image was randomly selected and included in the message, (2) the hashtag was randomly selected and included in the message if it did not already contain a hashtag, and (3) the URL parameter was replaced correctly. During the experiment, the application correctly used the message template text and substituted the message parameters 100% of the time as verified by both human reviewers and a custom algorithm using text search and attribute-matching techniques (Multimedia Appendix 5). The software code used to determine the correctness of the generated messages can be found on GitHub [36].

## Discussion

### Principal Findings

Our findings show that a software tool can support health promotion and education research by automating aspects of the generation, distribution, and assessment of hundreds of health promotion messages and user comments across different social media types with the highest degree of functional correctness and minimal human interaction. The detailed analysis of the messages and influence of several variables on user attention and engagement will be discussed in a forthcoming paper. We chose the 3 social media types, Twitter, Facebook, and Instagram, for the experiment because they were among the most popular social media platforms used by people living in the United States at the time of the experiment [11-13]. The software code is available on GitHub for free [36]. We invite readers and developers to use and develop the tool and to contribute to the development of evidence-based health promotion and interventions for social media.

The tool that we have presented here has the potential to support research teams and public health organizations. Research teams can use the tool to generate, manage, and test larger numbers of public health messages. The tool also contributes to standardizing social media research methods through 2 features: (1) it consistently applies randomization techniques to reduce selection bias (eg, message templates and images) and (2) the tool employs a data dictionary to contribute to more consistent reporting standards for social media research metrics (including clearly defined metrics and calculations such as click rate).

By surfacing toxic comments that may include offensive and disrespectful language and could negatively impact an online health conversation, the tool also provides support for public health organizations that need to manage digital public health campaigns about controversial topics such as smoking and related regulations and policies. This became evident when an antismoking regulation campaign by the Chicago Department of Public Health resulted in significant backlash by Twitter users, sending more than 600 tweets in 1 week against the proposed regulation [37]. The tool that we have described here may support public health organizations charged with the implementation of controversial health campaigns that may require monitoring and moderation of larger volumes of comments.

### Limitations of the Study and Tool

Here, we present the findings of a correctness analysis that was focused on assessing the probability with which a software tool generates and distributes correct health messages across different social media and collects message performance data and user comments. Trial Promoter focuses on social media–based campaigns. The distributed messages (organic messages and paid advertisements) would, therefore, not be viewed by individuals who do not use social media but may still be part of the targeted population of interest.

Social media also provides a method for reaching specific populations based on their characteristics (eg, age, gender, location, language, and interests). These targeting capabilities are usually built into the social media platforms and are based upon proprietary user data that are not available to the study team. Using this version of the tool, users need to set up the targeting on the social media platform, whereas Trial Promoter integrates with the social media platform to distribute the message content.

The current version of the code only supports integration with Twitter, Facebook, and Instagram, which were selected owing to their popularity in the United States. Researchers and health organizations in other parts of the world may also want to consider integrating other social media platforms with Trial Promoter.

Research teams might also require additional functionality to answer their specific research questions, for example, different types of randomizations, other social media platforms, ability to incorporate social media monitoring data, and mentions of social media influencers—all of which would need to be developed as extensions to the current version of the application.

Furthermore, additional features could be added in the future to enhance the application such as taking into account disease and health topic awareness months, trending topics and hashtags on social media, which may affect social media user attention and engagement, as well as the automatic blocking of social media users who contribute toxic comments, or automated debiasing of social media datasets using software programs such as BotOrNot [38].

Finally, future research will need to examine in more detail the effectiveness of social media–driven health promotion efforts to communicate risks and effects of harmful products and behaviors to promote healthy lifestyles and behaviors.

### Ethical and Data Privacy Considerations

As social media is designed to foster social interactions, health promotion and intervention campaigns on social media can lead to user comments that may include identifiable or personal health information, which poses privacy issues, safety risks, and dignitary violations [39]. Trial Promoter has the ability to display user comments received in response to a campaign and surface those comments that may require moderation owing to their toxicity (ie, offensive, disrespectful language that could negatively impact an online conversation). However, the tool does not directly moderate and, for example, delete specific comments or ban disruptive users on social media platforms. It is possible to disable comments on some social media, such as Facebook, as a measure to mitigate such risks. However, some social media, such as Twitter, do not allow the deletion of replies to a message. In this case, we suggest the use of disclaimer messages as suggested by Bender et al [16], for example, “Social media is not secure. Please don’t post if you are concerned about your privacy” *.*

In addition, the current version of the tool does not identify and moderate comments that may be nontoxic but still raise critical privacy and safety issues and may require a response. For example, a social media user may comment on a depression advertisement about their current and imminent suicidality or may leave a comment disclosing current child abuse on one of these advertisements. It is worthwhile to note that Facebook launched a suicide alert reporting system so that Facebook users can report individuals who they believe are expressing suicidal thoughts or intent [40,41]. To manage user comments using the current version of the tool, we suggest having a moderator who monitors user comments daily and manages them on a case-by-case basis.

Furthermore, Trial Promoter uses a number of third-party applications to support specific tasks, 2 of which may raise privacy concerns that researchers should be aware of. First, for the comment analysis, Trial Promoter shares the user comments (not the username or other information) with the third-party application, *Perspective*, developed by Jigsaw under the umbrella of *Google* ’s parent company, *Alphabet*. However, user comments are considered identifiable information. A recent study found that online searches of verbatim Twitter quotes found in journal articles can be traced back to individual users 84% of the time [42]. It is not possible to delete the comments after the analysis as the *Perspective* application falls under *Google* ’s privacy policy [43]: “The rights you grant in this license are for the limited purpose of operating, promoting, and improving our Services, and to develop new ones.” Although *Google* states that it does not share the content uploaded with third parties, which also limits potential conflict of interest, *Google* can use the comments submitted to the API to improve their machine learning model used for the analysis of the comments. We believe that as *Google* uses the data merely to improve its *Perspective* app and the dataset is not available publicly, the use of this third-party application is within the ethical and regulatory guidelines to protect users’ privacy. Second, our local version of the Trial Promoter tool is hosted by the cloud-based hosting provider, Heroku, a Salesforce application. Salesforce has passed security and privacy-related audits and certifications including the EU-US Privacy Shield Framework and TRUSTe Certification [15]. Any group or institution that decides to host Trial Promoter will have to ensure the privacy and security of their preferred hosting platform.

Finally, as we expand Trial Promoter, our team intends to address the current limitations of the tool as well as ethical issues such as privacy concerns, variations in protection across different platforms, and expectations of end users and other stakeholders by incorporating the *Hippocratic Oath* for technology, that is, a greater focus on the ethics of technology design [16]. We welcome collaborators.

### Comparison With Previous Work

To ensure the effective use of social media in research and to propose and assess evidence-based public health programs for social media, previous work has emphasized the need for flexible technical applications including Web-based data-gathering techniques that are readily available to research teams as well as consistent and transparent frameworks for data collection, quality assessment, debiasing techniques, and systematic reporting standards—most of which are currently lacking [15,41-44]. In addition, automated content generation and distribution for online use—in particular on social media—offers new possibilities for research and public health communities and could benefit the development and implementation of public health promotion efforts. Advanced applications, so-called *bots*, could generate and distribute information and, in some cases, interact with messages. They are *regarded an influential but also somewhat mysterious factor in public discourse and opinion making* [45]. However, a tool that supports research efforts in this field does not exist as yet. Previous research showed that a Twitter bot sharing public health information was perceived as *credible*, *attractive*, and *competent* [46]. These data suggest that bots could potentially be utilized by research and public health organizations. Additional work demonstrated that automatically generated content by a software application is perceived as descriptive and boring but also considered to be objective and not necessarily discernible from content written by journalists” [43]. Bots have been studied in a variety of contexts (eg, prosmoking and protobacco campaigns [15,44], activism or advocacy [47], social networks and human communication decisions [48,49], social shaping [50], content pollution [51], social metric gaming [52], ranking manipulation [53], infiltration [54], political astroturfing [55], recommendation [56], scholarship dissemination [57], and journalism [58]). However, there are little data on bot-like applications that would benefit health promotion research and the development of health communication interventions.

We do not suggest that the tool described here is a bot because the content that makes up the messages needs to be selected and imported by a human actor and the application does not mimic humans and/or human behavior [59], that is, it does not act as an automated social actor similar to how humans might act in social spaces [49,51]. That said, the authors are not aware of similar research that has developed and tested a tool for automatic postings of public health messages on social media to enable better health promotion and intervention research in the digital age. Further studies on automatically generated social media content will help to better understand its role in supporting the public health agenda and health promotion research.

### Conclusions

The tool (Trial Promoter) that we have presented here has the potential to influence social media–enabled health promotion and intervention research and practice. First, it enables the assessment of large numbers of messages to develop evidence-based communication approaches for social media. This is especially important as the use of social media among US adults varies by demographic characteristics such as age, gender, and race and ethnicity, and across social media [11-13]. Thus, the success of digital health communication efforts might not only depend on the type of content (eg, text, image, audio, and video) but also on other variables such as social media type, organic versus paid (advertisements) medium, the message date and time, and user’s social networks. The tool presented here offers a way of assessing the influence of these variables on the effectiveness of social media–based health promotion and intervention efforts. Second, the tool can be used by public health organizations to increase their output of health education messages, for example, to potentially counteract the growing prevalence of online marketing featuring products and behaviors harmful to health, for example, tobacco products and drugs [57-59]. Finally, the tool also assists with identifying and moderating larger volumes of user comments to the distributed messages. The tool surfaces those comments that may include offensive, disrespectful language and could negatively impact an online conversation. We call on readers and developers to use and further develop the software code and to contribute to the development of evidence-based health communication approaches in the digital age.

## Appendix

Multimedia Appendix 1 Example of data validation. The screenshot shows results for a resolution check of images to ensure they meet the requirements of the social media platform Instagram. The row highlighted in red indicates that the image does not meet the requirements for square pictures of a minimum of 600x600 pixels. Images shown here are samples similar to the original copyright protected campaign images. Images are courtesy of Dragqueen (top), Edwin Ortiz (middle), Alex Proimos (bottom) at commons.wikimedia.org.

Multimedia Appendix 2 Screenshot of experiment parameter setup form.

Multimedia Appendix 3 Third-party applications used for collecting key performance data.

Multimedia Appendix 4 Example of a data dictionary used by the application.

Multimedia Appendix 5 Screenshot of interface that shows the results of the automated correctness analysis performed by the application.

## Abbreviations

API: Application Programming Interface
CSV: Comma Separated Values
FDA: Food and Drug Administration
JSON: JavaScript Object Notation
NCI: National Cancer Institute
NIH: National Institutes of Health
REST: Representational State Transfer
