# Supplementary figures and images for: A Software Tool Aimed at Automating the Generation, Distribution, and Assessment of Social Media Messages for Health Promotion and Education Research
Source: JMIR Public Health Surveill. 2019 May 7;5(2):e11263. doi: 10.2196/11263 (PMC6528439; doi:10.2196/11263)

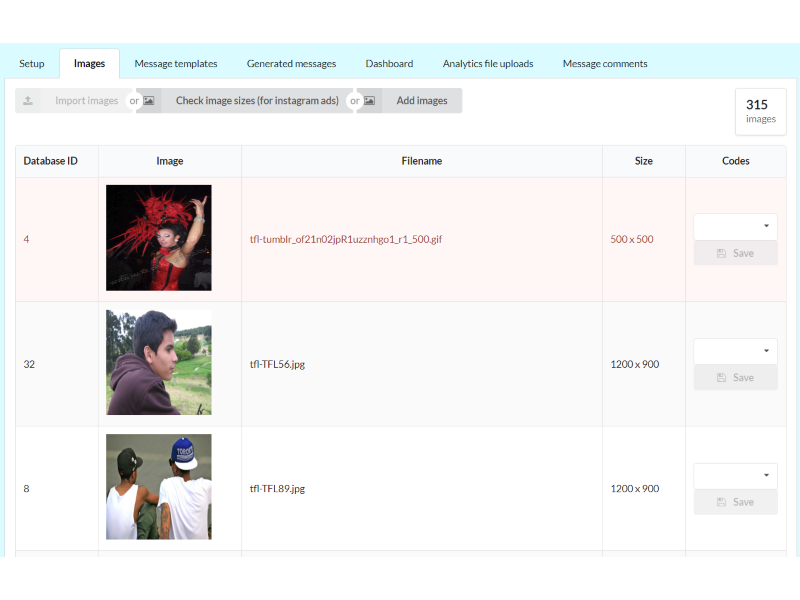

Supplement: Multimedia Appendix 1 [file publichealth_v5i2e11263_app1.png]

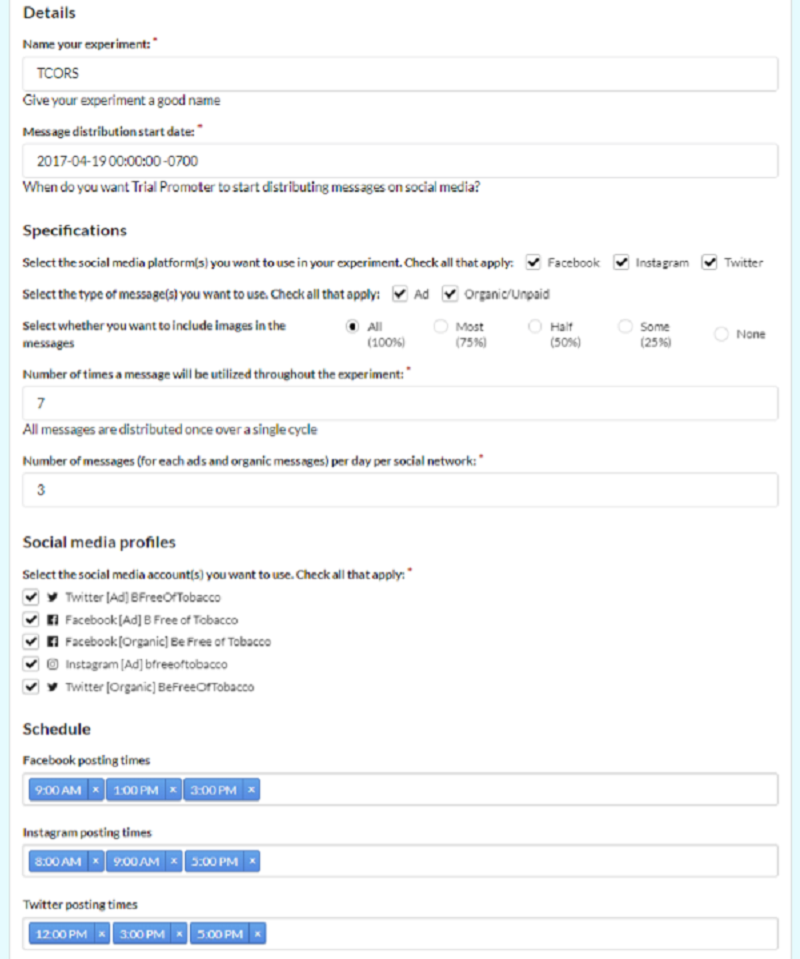

Supplement: Multimedia Appendix 2 [file publichealth_v5i2e11263_app2.png]

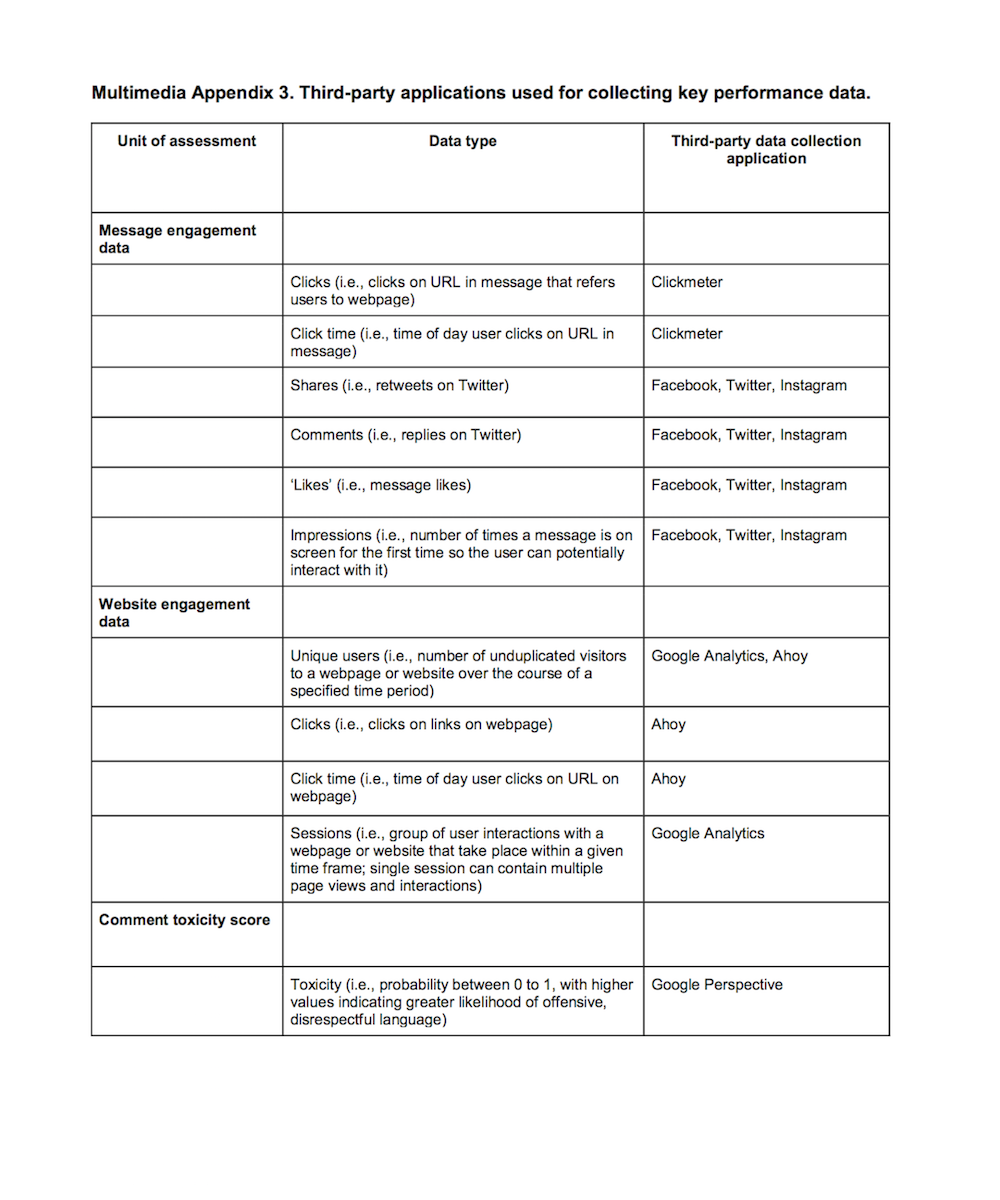

Supplement: Multimedia Appendix 3 [file publichealth_v5i2e11263_app3.png]

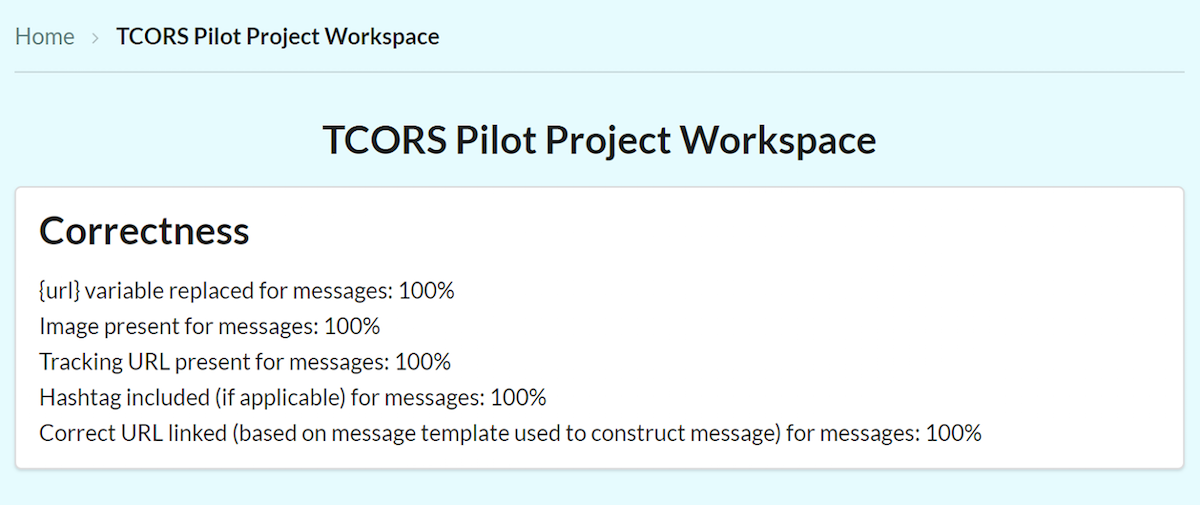

Supplement: Multimedia Appendix 5 [file publichealth_v5i2e11263_app5.png]
